# Comparison of combined suprascapular and axillary nerve pulsed radiofrequency and nerve block for the treatment of primary frozen shoulder: a prospective cohort study

**DOI:** 10.1080/07853890.2025.2456692

**Published:** 2025-02-04

**Authors:** Yue Wu, Jiangyou Huang, Weibo Zhang, Suming Tian, Gang Chen

**Affiliations:** a Department of Anesthesia and Pain Management, Sir Run Run Shaw Hospital, School of Medicine, Zhejang University, Hangzhou, Zhejiang, China; b Department of Anesthesia and Pain Management, Hangzhou Chengdong Hospital, Hangzhou, Zhejiang, China

**Keywords:** Frozen shoulder, shoulder pain, suprascapular nerve, axillary nerve, pulsed radiofrequency

## Abstract

**Objectives:**

To compare the effects of pulsed radiofrequency (PRF) and block of the suprascapular nerve (SSN) and axillary nerve (AN) in patients with primary frozen shoulder (FS).

**Methods:**

Patients with primary FS received PRF (Group P) or block (Group B). Shoulder pain during rest, activity and sleep was measured by a numerical rating scale (NRS), the Shoulder Pain and Disability Index (SPADI) was used to assess shoulder function and disability, and the passive range of motion (PROM) of the shoulder joint was measured by a digital inclinometer. Assessments were made at baseline and 2 weeks and 1, 3, and 6 months after the procedure.

**Results:**

Of the 74 patients, 63 were eventually included, and a total of 3 patients were lost to follow-up. Finally, 60 patients (30 in each group) completed the final analysis. There was a significant improvement in all outcome measures from baseline to 6 months after the procedure. Compared with those in group B, the NRS scores during activity and sleep in group P decreased more at 6 months after the procedure (*p* = 0.005 and 0.028). SPADI total scores were lower at 3 and 6 months after the procedure (*p* = 0.021 and 0.001). At different time after the procedure, most of the parameters of PROM improved more in group P than those in group B (flexion at 3 and 6 months, *p* = 0.042 and <0.001; abduction at 3 and 6 monthse, *p* = 0.001 and 0.001; extension at 3 and 6 months, *p* = 0.038 and 0.007, internal rotation at 6 months, *p* = 0.015; external rotation at 1, 3, and 6 months, *p* = 0.002, 0.002, and 0.001, respectively).

**Conclusions:**

In patients with primary FS who completed both manipulation under anesthesia and intra-articular injections, PRF with SSN and AN appears to provide better pain relief, better PROM recovery, and more shoulder function improvement than nerve block treatment.

## KEY MESSAGES

Pulsed radiofrequency and blocking of the suprascapular nerve and axillary nerve have become new treatment options for primary frozen shoulder.

This study showed that pulsed radiofrequency delivery via the suprascapular nerve and axillary nerve appears to provide better pain relief, better PROM recovery, and more shoulder function improvement than nerve block treatment.

## Introduction

Frozen shoulder (FS) is also known as shoulder adhesive capsulitis and is a shoulder disease characterized by progressive pain and loss of active and passive mobility of the glenohumeral joint; FS significantly affects patients’ quality of life and imposes significant financial and medical burdens. FS is most common in women aged 40–60 years [1], and its prevalence in the general population is between 2% and 5% [2,3]; however, its prevalence in patients with diabetes and thyroid disease is as high as 38% [4].

The specific etiology of primary FS is unknown. FS typically progresses through three stages: the freezing, frozen and thawing phases [5]. During the frozen phase, except for shoulder pain, joint movement is obviously limited, the surrounding soft tissues are in a “frozen” state, and atrophy of the supraspinatus, infraspinatus and deltoid muscles may occur [6]. This stage has a significant impact on patients’ daily activities and sleep.

Although FS is considered a self-limiting disorder, its duration remains unclear [7]. Hand C et al. [8] conducted long-term follow-up of 223 patients with primary FS for 2 to 20 years (average 4.4 years) and reported that 35% of patients still had mild-to-moderate pain and that 6% of patients had severe pain and dysfunction. There is still no evidence that FS heals naturally without treatment [7]. Therefore, active and standardized treatment is crucial, and early pain relief, maximum improvement in shoulder joint function and improvement in the quality of life are the main treatment goals for FS [9].

FS treatment includes oral nonsteroidal anti-inflammatory analgesics, physical therapy, intra-articular (IA) injection of corticosteroids, manipulation under anesthesia (MUA), arthroscopic capsular release (ACR), and rehabilitation exercise [10]. There is still no consensus on the optimal treatment strategy for FS, and even with the above systematic and standardized treatment, the effect is still limited, and the recovery is slow [10].

The use of nerve block as a treatment option for shoulder pain has received increasing amounts of attention in recent years [11]. The peripheral nerve innervation of the shoulder joint is highly complex, but the suprascapular nerve (SSN) and the axillary nerve (AN) play important roles in the innervation of the glenohumeral joint [12]. The SSN provides sensation to the posterior and upper regions of the glenohumeral joint, while the AN innervates its anterior and lower regions [13].

The glenohumeral joint innervated by the above nerves is rich in autonomic nerve fibers, which easily cause reflex vasculature disorders due to inflammatory stimulation and form a vicious cycle of “pain - ischemia - pain”. However, combined SSN and AN block can interrupt this vicious cycle, cut off the conduction pathway of the pain reflex, relieve shoulder vasospasm, improve local blood flow, reduce inflammation and promote pain relief.

However, the duration of nerve block treatment is relatively short [14], so there is an urgent need for more durable treatment. In recent years, pulsed radiofrequency (PRF) has been developed as a new technique for the treatment of pain. In contrast to traditional radiofrequency (RF) thermocoagulation, which involves an intermittent RF current, the temperature generated is not greater than 42 °C, and this can render the pain conduction fiber inactive without damaging the anatomical structure of the nerve fiber. PRF was not shown to cause decreased muscle strength or skin sensory disorders, and although it may cause adverse events or complications, including vascular injury, bleeding, hypotension, infection, etc., standard and skilled operation makes it extremely rare [15].

However, whether PRF of the SSN and AN can achieve better therapeutic effects than nerve block in the treatment of primary FS has not been reported in the literature. Therefore, the purpose of this study was to investigate and compare the effects of PRF and block of the SSN and AN on pain relief and improvement of shoulder function in patients with FS.

## Materials and methods

### Study design

This was a prospective cohort study, and the protocol was approved by the ethics review committee of Sir Run Run Shaw Hospital, School of Medicine, Zhejiang University (approval no. 20200525-28), and the clinical trial registry in China (https://www.chictr.org.cn) was used to complete the registration (no. ChiCTR2200057456). The study was conducted in accordance with the 1964 Declaration of Helsinki (as amended in 2013). Patients with primary FS who underwent PRF or blockage of the SSN and AN at Sir Run Run Shaw Hospital, School of Medicine, Zhejiang University between March 12, 2022, and February 20, 2023, were included in the study. X-ray and magnetic resonance imaging (MRI) of shoulder joints were routinely completed before all patients were enrolled in the study. Written informed consent was obtained from all participants prior to inclusion. The inclusion criteria for this study were as follows: (1) met the clinical diagnostic criteria for primary FS [16] (Table 1); (2) were aged ≥18 years; and (3) had an NRS score ≥4 for pain during shoulder movement on the affected side. The exclusion criteria were as follows: (1) had severe cardiopulmonary insufficiency or mental illness; (2) a history of shoulder surgery on the affected side or cervical radiculopathy or other chronic pain; (3) MRI evidence of tendon rupture in the shoulder; (4) abnormal platelet and coagulation function; (5) a history of malignancy, stroke, diabetes or thyroid disease; and (6) refusal to complete the questionnaire or inability to be contacted during follow-up.

**Table 1.** Clinical diagnostic criteria for frozen shoulder [16].

Shoulder pain.
Limited range of motion of shoulder joint (forward flexion, abduction, internal and external rotation).
X-rays excluded other shoulder lesions, such as advanced shoulder osteoarthritis, pathological fracture, dislocation, avascular necrosis, and calcifying rotator cuff tendinopathy.
MRI findings of coracohumeral ligament thickening, rotator interval infiltration of the subcoracoid fat, and axillary recess thickening yield high specificity.

All procedures were performed by the same experienced doctor and guided by ultrasound (Mindray, Bothell, Shenzhen, China). The subjects were asked to lie on their side on the operating table with the affected side facing up and a thin pillow under their head. ECG monitoring was performed throughout the whole process, and the SSN and AN puncture operations were performed according to the methods described by Esparza-Minana JM et al. [17].

A 22-gauge RF electrode trocar (InnoMand Medical Technology Co., Ltd., Emmendingen, Germany) with a length of 15 cm and a working end of 10 mm was applied to the affected shoulder after disinfection and local anesthesia. First, the puncture target was the SSN, and a low-frequency convex array probe (2-5 MHz, musculoskeletal mode) was placed laterally on the affected side of the scapular ganglion in the oblique coronal position.

**Figure 1.**
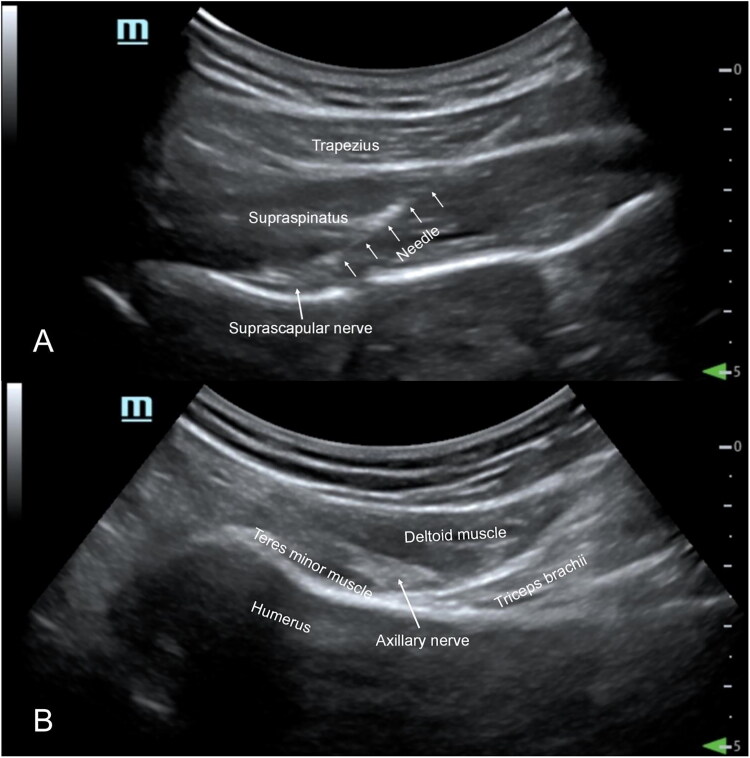
Ultrasound-guided puncture of SSN (A) and an (B). Note: SSN, suprascapular nerve; AN, axillary nerve.

By tilting the probe forward, the trapezius muscle, supraspinatus muscle and deep supraspinatus fossa can be identified *via* ultrasonic imaging. The suprascapular artery and vein were identified by color Doppler flow, and the SSN accompanied them. The in-plane technique was used to insert the needle. The needle tip reached the supraspinatus fossa and was close to the blood vessel (Figure 1). The RF electrode was connected to complete the sensory and motor tests of the target nerve. When the following parameters were used (50-100 Hz, 1 ms pulse width, 0.3-0.5 V), the patient reported significant shoulder paresthesia; when the following parameters were used (2 Hz, 1 ms pulse width, 0.3-0.5 V), the supraspinatus and infraspinatus muscles were stimulated to twitch. After the accurate location was determined, the SSN was treated with PRF or blocked. Group P was administered PRF for 360 s (parameter: 2 Hz, 30 ms pulse width, 42 °C) to the SSN, while group B was administered an injection of 5 ml of drug mixture (configuration as follows: compound betamethasone injection [each bottle (1 ml)] contained 5 mg of betamethasone dipropionate and 2 mg of betamethasone disodium phosphate; Shanghai Schering-Plow Pharmaceutical Co., Ltd., Shanghai, China]; 1 ml + 2% lidocaine [Hunan Kelun Pharmaceutical Co., Ltd., Yueyang, Hunan, China]; and 5 ml + normal saline [Otsuka Pharmaceutical Co., Ltd., Tianjin, China] (9 ml).

The next puncture target was the AN, and the low-frequency convex array probe (2-5 MHz, musculoskeletal mode) was vertically placed behind the upper arm near the quadrilateral foramen. When the angle of the probe is adjusted, the posterior brachial circumflex artery can be observed in the ultrasound image between the teres minor, the deltoid and the triceps, which are identified by color Doppler flow. The needle was inserted vertically by an out-of-plane technique, and the tip of the needle reached the fascia space beside the posterior humeral circumflex artery (Figure 1). Sensory tests (patients reported paresthesia in the shoulder and upper arm regions) and motor tests (patients with deltoid twitches) were performed in the same way as described above. PRF treatment or block was applied to the AN in the same manner as for the SSN.

Both groups underwent IA injection of the glenohumeral joint and MUA once each. MUA is a method for releasing the attached shoulder joint capsule manually and can quickly and effectively improve the mobility of the shoulder joint [18]. The sequence was as follows: brachial plexus anesthesia *via* the intermuscular groove approach was performed first (local anesthetic combination: 2% lidocaine 5 ml + 0.75% ropivacaine [AstraZeneca AB, Sweden] 5 ml), followed by articular injection *via* the posterior glenohumeral approach (drug combination: 5 ml), using the same method as that used by Cho CH et al. [19]. After the shoulder anesthesia on the affected side had fully affected the patient, manipulation of the shoulder joint was performed. The specific method was the same as that described by Kraal T et al. [18].

**Figure 2.**
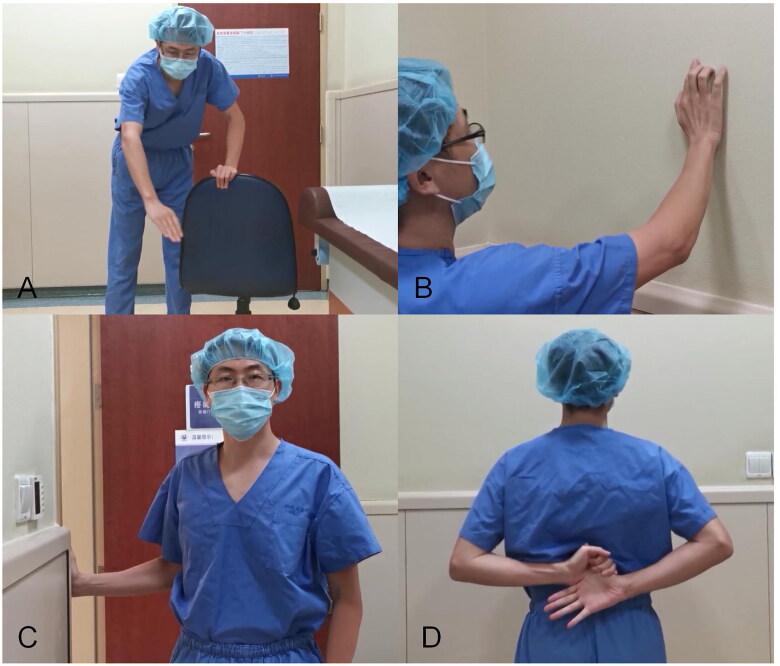
Home rehabilitation exercises. Note: A, pendulum exercises; B, wall-climbing exercises; C, passive external-rotation stretches; D, internal-rotation stretches.

All the subjects were instructed to perform home rehabilitation exercises to increase their range of motion; these exercises included pendulum exercises, wall-climbing exercises, passive external-rotation stretches and internal-rotation stretches, and they were performed three times a day (15 min each time) [3], the exercises in Figure 2 was demonstrated by the first author, Dr. Yue Wu, with his written statement of consent for publication. The participants were regularly contacted and monitored by the follow-up staff to ensure that they completed the home exercise as needed. The endpoint of follow-up was 6 months after the procedure. The data were collected by trained investigators at baseline (0) and 2 weeks (2w), 1 month (1 m), 3 months (3 m), and 6 months (6 m) postprocedure *via* outpatient visits.

### Outcome measures

The primary outcome was the degree of pain in the affected shoulder at rest, during activity, and during sleep at night, as measured by the Numerical Rating Scale (NRS), which is an 11-point scale ranging from 0 (no pain) to 10 (as much pain as you can imagine) [20].

The secondary outcomes were shoulder function and PROM. Shoulder joint function was assessed by the Shoulder Pain and Disability Index (SPADI) scale, which is divided into two sections: pain (5 items) and disability (8 items). Thirteen questions were answered on a 10-point scale, and the total SPADI score was calculated by adding all 13 items together and dividing by 130 (the highest score) multiplied by 100 [21]. The ROM, including flexion, abduction, extension, external rotation and internal rotation, was measured by a digital inclinometer (Model SYJDC200, Deqing Shengtaixin Electronic Technology Co., LTD., Huzhou, China). In addition, procedure-related adverse reactions and complications, including bleeding, infection, puncture pain, pneumothorax, local anesthetic intoxication reactions, fractures, dislocations, rotator cuff tears, were evaluated.

### Sample size calculation

According to a previous study [22], compared with physical therapy alone, PRF in the SSN has a better effect on shoulder pain in patients with adhesive capsulitis (1.7 ± 1.5 vs. 3.3 ± 2.5). A bilateral t test was used (α = 0.05), and the detection efficacy power (1-β) was 80%. It was assumed that PRF from the SSN and AN in during the treatment of primary FS in this study could achieve similar effects, and 28 patients were needed in each group. G-power software was used to determine the sample size.

### Statistical analysis

All the data were analyzed using SPSS 23 (IBM SPSS, Chicago, IL, USA). Normality was tested using the Shapiro‒Wilk test. Continuous data with a normal distribution are expressed as the mean (standard deviation), and categorical variables are expressed as numbers and percentages. Independent sample t tests or Mann‒Whitney U tests were used to compare the two groups. For classification parameters, chi-square analysis and Fisher’s exact test were used where appropriate. P values < 0.05 were considered to indicate statistical significance.

## Results

### Patient characteristics

**Figure 3.**
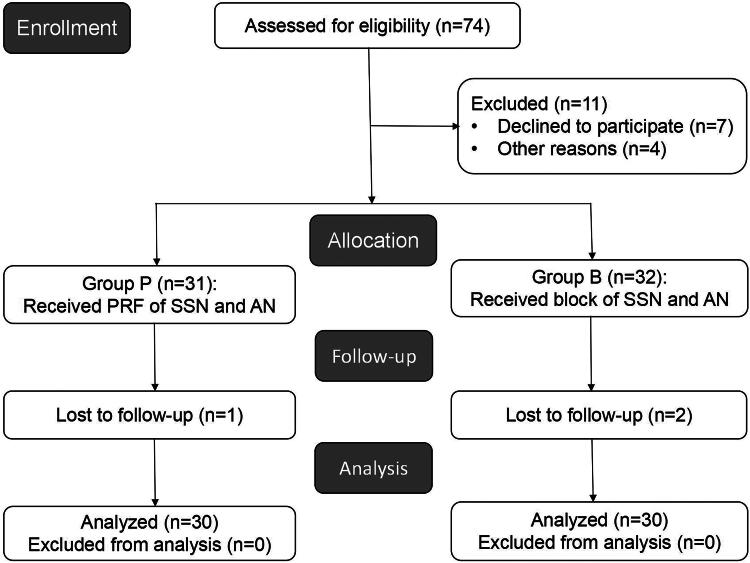
Consort flowchart of patient recruitment. Note: PRF, pulsed radiofrequency; SSN, suprascapular nerve; AN, axillary nerve.

From March 2022 to March 2023, a total of 74 subjects were assessed for eligibility, and 63 were eventually included (Figure 3). Seven patients refused to participate, among whom 3 patients completed PRF and 4 patients completed block. Among the 4 patients excluded for other reasons, 2 patients were complicated with supraspinatus tendon fracture (completed PRF, no MUA), 1 patient had a history of diabetes (completed PRF, no steroid injection), and 1 patient had thyroid disease (completed block). A total of three patients were lost during the follow-up period; two patients (one each in groups P and B) could not be contacted 1 and 3 months after the procedure, and one patient underwent cervical surgery due to acute cervical trauma; this was thus interrupted 3 months after the procedure. Ultimately, 60 patients with the following characteristics were included in the analysis: 54.38 ± 7.89 years old, a BMI of 23.80 ± 3.18 kg/m^2^, 44 females (73.3%), 31 patients (51.7%) with left shoulder pain, and a pain duration of 7.08 ± 3.35 months. The baseline data, including demographic and clinical characteristics, were not significantly different between the two groups (30 patients each) (*p* > 0.05) (Table 2).

**Table 2.** Demographic and clinical characteristics of patients in two groups.

Parameter	Group P (*N* = 30)	Group B (*N* = 30)	Total (*N* = 60)	*P* value
**Age (years)**	53.67 ± 8.74	55.10 ± 7.00	54.38 ± 7.89	0.486
**Sex, n (%)**				
Female	20(66.7)	24 (80.0)	44 (73.3)	0.243
Male	10 (33.3)	6 (20.0)	16 (26.7)	
**Height (cm)**	161.53 ± 7.98	160.97 ± 7.62	161.25 ± 7.74	0.779
**Weight (kg)**	63.03 ± 10.11	60.93 ± 10.10	61.98 ± 10.08	0.424
**BMI (kg/m^2^)**	24.16 ± 3.49	23.45 ± 2.85	23.80 ± 3.18	0.390
**Affected side, n (%)**				
Left	17 (56.7)	14 (46.7)	31 (51.7)	0.438
Right	13 (43.3)	16 (53.3)	29 (48.3)	
**Dominant extremity, n (%)**				
Left	3 (10.0)	2 (6.7)	5 (8.3)	0.640
Right	27 (90.0)	28 (93.3)	55 (91.7)	
**Duration of symptoms (Months)**	7.20 ± 3.35	6.97 ± 3.41	7.08 ± 3.35	0.790
**Comorbidities, n (%)**				
Hypertension	10 (33.3)	7 (23.3)	17 (28.3)	0.390
Pulmonary disease	1 (3.3)	2 (6.7)	3 (5.0)	0.554
Coronary heart disease	3 (10.0)	4 (13.3)	7 (11.7)	0.688
Hypercholesterolemia	5 (16.7)	4 (13.3)	9 (15.0)	0.718
Rheumatoid arthritis	1 (3.3)	0 (0)	1 (1.7)	0.313
**Previous treatment history, n (%)**				
NSAIDs use	16 (53.3)	13 (43.3)	29 (48.3)	0.438
Steroid injection	11 (36.7)	9 (30.0)	20 (33.3)	0.584
Physiotherapy	11 (36.7)	15 (50.0)	26 (43.3)	0.297
Acupuncture	5 (16.7)	7 (23.3)	12 (20.0)	0.519

NOTE. Data are presented as mean (standard deviation) or number of patients (%); Group P, Group of PRF for SSN and AN; Group B, Group of nerve block for SSN and AN; SSN, suprascapular nerve; AN, axillary nerve; PRF, pulsed radiofrequency; BMI, body mass index; NSAIDs, nonsteroidal anti-inflammatory analgesics.

### Efficacy of treatment

Compared with that before the procedure, the affected shoulder pain, especially movement and nocturnal pain, was significantly relieved at different follow-up times after the procedure (*p* < 0.01). There was no significant difference in resting pain between the two groups (*p* ≥ 0.05). There was no difference in movement pain or nocturnal pain between the two groups at 3 months after the procedure, and the NRS scores in group P were lower than those in group B at 6 months after the procedure (*p* = 0.005 and 0.028) (Table 3).

**Table 3.** Changes of shoulder pain on the affected side.

	Group P (*N* = 30)		Group B (*N* = 30)		
Parameter	Mean ± SD	*P* value	Mean ± SD	*P* value	*P* value
**Resting pain**					
Baseline	2.27 ± 0.64		2.53 ± 0.78		0.152
2 w	1.37 ± 0.56	<0.001^#^	1.43 ± 0.63	<0.001^#^	0.664
1 m	1.07 ± 0.69	<0.001^#^	1.33 ± 0.71	<0.001^#^	0.146
3 m	0.80 ± 0.55	<0.001^#^	0.90 ± 0.55	<0.001^#^	0.484
6 m	0.63 ± 0.66	<0.001^#^	0.70 ± 0.70	<0.001^#^	0.708
**Movement pain**					
Baseline	6.97 ± 1.13		7.03 ± 0.96		0.807
2 w	3.50 ± 1.01	<0.001^#^	3.73 ± 0.98	<0.001^#^	0.367
1 m	2.97 ± 0.81	<0.001^#^	3.33 ± 1.09	<0.001^#^	0.145
3 m	2.80 ± 0.76	<0.001^#^	3.03 ± 0.96	<0.001^#^	0.303
6 m	1.40 ± 0.72	<0.001^#^	1.97 ± 0.76	<0.001^#^	0.005^#^
**Nocturnal pain**					
Baseline	5.07 ± 0.74		5.13 ± 0.82		0.742
2 w	1.90 ± 0.88	<0.001^#^	2.17 ± 0.79	<0.001^#^	0.224
1 m	1.53 ± 0.78	<0.001^#^	1.70 ± 0.70	<0.001^#^	0.387
3 m	1.43 ± 0.73	<0.001^#^	1.47 ± 0.57	<0.001^#^	0.844
6 m	1.00 ± 0.64	<0.001^#^	1.37 ± 0.61	<0.001^#^	0.028*

NOTE. Data are presented as mean (standard deviation); Group P, Group of PRF for SSN and AN; Group B, Group of nerve block for SSN and AN; SSN, suprascapular nerve; AN, axillary nerve; PRF, pulsed radiofrequency; Baseline, before the procedure; 2 W, 2 weeks after the procedure; 1 M, 1 month after the procedure; 3 M, 3 months after the procedure; 6 M, 6 months after the procedure.

* Significant difference between the two groups or within groups at *p < .05; #Significant difference between the two groups or within groups at.

^#^ *p* < .01.

**Figure 4.**
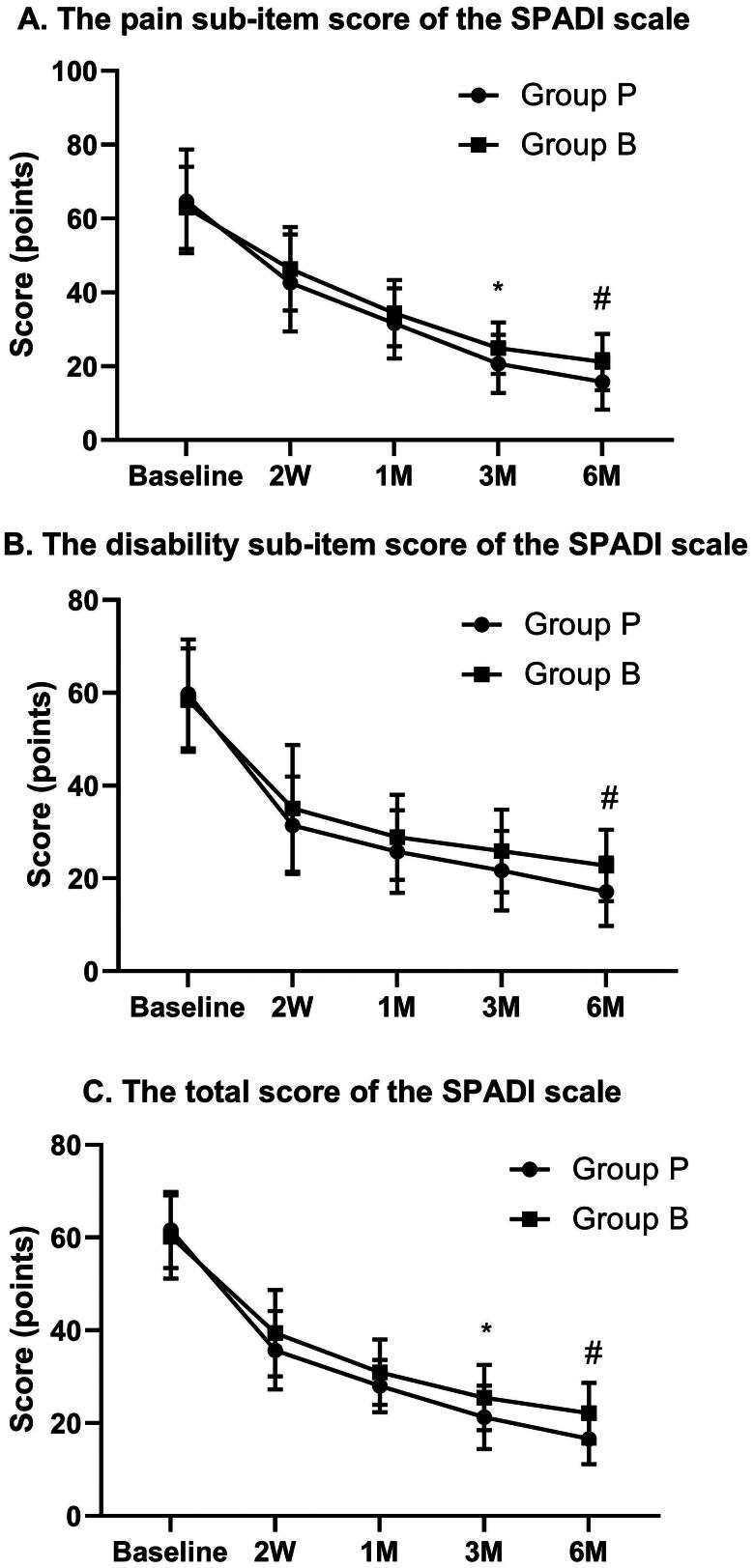
The changes of the pain, disability sub-item and total SPADI scale scores at different follow-up times. NOTE. Data are presented as mean (standard deviation); Group P, Group of PRF for SSN and AN; Group B, Group of nerve block for SSN and AN; SSN, suprascapular nerve; AN, axillary nerve; PRF, pulsed radiofrequency; SPADI, the shoulder pain and disability index; Baseline, before the procedure; 2 W, 2 weeks after the procedure; 1 M, 1 month after the procedure; 3 M, 3 months after the procedure; 6 M, 6 months after the procedure. *Significant difference between the two groups at *P < .05. #Significant difference between the two groups at *P < .01.

Moreover, the pain, disability and total SPADI scores improved significantly at different follow-up times after the procedure (*p* < 0.01). The pain and total SPADI scores in group P were lower than those in group B at 3 and 6 months after the procedure (pain SPADI, 20.67 ± 7.85 & 24.93 ± 6.96 [*p* = 0.030] and 15.78 ± 7.53 & 21.20 ± 7.62 [*p* = 0.008]; total SPADI, 21.28 ± 6.84 & 25.53 ± 7.01 [*p* = 0.021] and 16.61 ± 5.44 & 22.17 ± 6.56 [*p* = 0.001]). The disability score on the SPADI decreased more in group P than in group B at 6 months after the procedure (17.13 ± 7.39 & 22.79 ± 7.70 [*p* = 0.005]) (Figure 4).

The different parameters of PROM after the procedure were significantly improved compared with those before the procedure (*p* < 0.01). At 3 and 6 months after the procedure, patients in group P experienced greater increases in flexion, abduction, and extension than did those in group B (flexion, 149.17 ± 18.06 & 140.17 ± 15.40 [*p* = 0.042] and 159.33 ± 15.80 & 143.50 ± 13.66 [*p* < 0.001]; abduction, 146.53 ± 17.21 &130.33 ± 19.69 [*p* = 0.001] and 158.00 ± 19.24 & 139.17 ± 20.39 [*p* = 0.001]; extension, 49.50 ± 7.35 & 45.33 ± 7.87 [*p* = 0.038] and 51.33 ± 6.56 & 46.33 ± 7.30 [*p* = 0.007], respectively); compared with patients in group B, those in group P experienced greater increases in internal rotation at 6 months after the procedure (66.80 ± 8.08 & 61.50 ± 8.22 [*p* = 0.015]); at 1, 3, and 6 months after the procedure, group P experienced greater increases in external rotation than did group B (55.00 ± 7.31 & 62.00 ± 8.96 [*p* = 0.002], 57.33 ± 8.68 & 65.50 ± 10.70 [*p* = 0.002], and 57.83 ± 9.26 & 67.17 ± 10.80 [*p* = 0.001], respectively) (Table 4).

**Table 4.** Changes of joint range of motion in the two groups.

	Group P (*N* = 30)		Group B (*N* = 30)		
Parameter	Mean ± SD	*P* value	Mean ± SD	*P* value	*P* value
**Flexion**					
** Baseline**	98.77 ± 25.28		97.03 ± 22.89		0.782
** 2 W**	130.60 ± 21.81	<0.001^#^	124.43 ± 18.07	<0.001^#^	0.238
**1 M**	140.33 ± 18.24	<0.001^#^	137.33 ± 15.52	<0.001^#^	0.495
**3 M**	149.17 ± 18.06	<0.001^#^	140.17 ± 15.40	<0.001^#^	0.042*
**6 M**	159.33 ± 15.80	<0.001^#^	143.50 ± 13.66	<0.001^#^	<0.001^#^
**Abduction**					
** Baseline**	80.60 ± 18.06		84.07 ± 19.04		0.472
**2 W**	105.17 ± 19.14	<0.001^#^	104.17 ± 17.43	<0.001^#^	0.833
**1 M**	132.33 ± 18.60	<0.001^#^	123.00 ± 18.63	<0.001^#^	0.057
**3 M**	146.53 ± 17.21	<0.001^#^	130.33 ± 19.69	<0.001^#^	0.001^#^
**6 M**	158.00 ± 19.24	<0.001^#^	139.17 ± 20.39	<0.001^#^	0.001^#^
**Extension**					
** Baseline**	32.20 ± 7.88		31.83 ± 7.60		0.855
**2 W**	38.17 ± 7.71	<0.001^#^	36.83 ± 7.48	<0.001^#^	0.499
**1 M**	43.00 ± 7.38	<0.001^#^	40.17 ± 7.59	<0.001^#^	0.148
**3 M**	49.50 ± 7.35	<0.001^#^	45.33 ± 7.87	<0.001^#^	0.038*
**6 M**	51.33 ± 6.56	<0.001^#^	46.33 ± 7.30	<0.001^#^	0.007^#^
**Exteral rotation**					
** Baseline**	18.87 ± 9.05		19.40 ± 9.26		0.822
**2 W**	42.50 ± 9.17	<0.001^#^	45.33 ± 10.17	<0.001^#^	0.262
**1 M**	55.00 ± 7.31	<0.001^#^	62.00 ± 8.96	<0.001^#^	0.002^#^
**3 M**	57.33 ± 8.68	<0.001^#^	65.50 ± 10.70	<0.001^#^	0.002^#^
**6 M**	57.83 ± 9.26	<0.001^#^	67.17 ± 10.80	<0.001^#^	0.001^#^
**Internal rotation**					
** Baseline**	17.53 ± 7.71		16.20 ± 7.24		0.493
**2 W**	50.17 ± 9.78	<0.001^#^	49.53 ± 8.18	<0.001^#^	0.787
**1 M**	60.00 ± 8.09	<0.001^#^	57.00 ± 7.38	<0.001^#^	0.139
**3 M**	63.30 ± 8.34	<0.001^#^	59.17 ± 8.41	<0.001^#^	0.061
**6 M**	66.80 ± 8.08	<0.001^#^	61.50 ± 8.22	<0.001^#^	0.015*

NOTE. Data are presented as mean (standard deviation); Group P, Group of PRF for SSN and AN; Group B, Group of nerve block for SSN and AN; SSN, suprascapular nerve; AN, axillary nerve; PRF, pulsed radiofrequency; Baseline, before the procedure; 2 W, 2 weeks after the procedure; 1 M, 1 month after the procedure; 3 M, 3 months after the procedure; 6 M, 6 months after the procedure; *Significant difference between the two groups or within groups at.

* *p* < .05;.

^#^ Significant difference between the two groups or within groups at ^#^*p* < .01.

### Adverse reactions and complications

Five patients in group P and seven patients in group B reported mild pain during injection (NRS 2-3) that was not specifically managed. No serious adverse reactions or complications, such as bleeding, infection, pneumothorax, tendon rupture, joint dislocation or fracture, were found during the operation or follow-up.

## Discussion

FS is characterized by restriction of passive and active shoulder joint movement and associated pain and is associated with chronic inflammation of the subsynovial membrane, which leads to thickening, fibrosis, contracture, and loss of the normal axillary recess [23]. Pathologically, the collagen structure of the shoulder joint capsule is replaced by hyperplastic tissue fibrosis and adjacent synovial thickening, accompanied by the growth of new vessels and nerves and inflammation, resulting in reduced shoulder joint volume, increased joint capsule stiffness, and eventually pain and limited joint activity [5].

FS was considered a self-limiting disease lasting 1-3 years. Even after a series of systematic treatments, 20%-50% of patients still experience pain and joint dysfunction, which shows that treatment is actually a substantial challenge, and the traditional theory of FS is also subject to an increasing number of disputes [8,24]. Treatment based on that theory is not generally recognized as the most effective treatment for FS [25].

The goal of FS treatment is to reduce pain and restore range of motion. In clinical work, we are accustomed to using a variety of methods for the treatment of FS, including IA injections of corticosteroids, MUA and rehabilitation exercise. IA injections can significantly reduce pain and improve joint function in FS patients, but these results are mainly in the short term [26,27]. In a randomized, double-blind, controlled study, IA injection of corticosteroids was compared with sham injection in FS, and it was found that patients receiving IA injection experienced significant pain relief after 6 weeks, but the effect was only maintained for 12 weeks [28]. MUA is a simple procedure for removing capsular adhesions and improving the range of motion of joints in a short period. Studies [29,30] compared MUA and ACR and followed them up for 1 year. MUA was found to have a significant effect on reducing shoulder pain and improving shoulder joint function score and ROM, but its effect was not significantly different from that of ACR. However, MUA has a greater cost effect. In this study, MUA was used in two groups of FS patients to achieve joint synergy between different treatment approaches. After the above combined treatment with active rehabilitation training at home, we strived to achieve better functional recovery of the shoulder joint [31].

Even through these comprehensive treatment strategies are used, the treatment efficacy is still poor for some patients. To achieve better treatment effects, the shoulder peripheral nerve is used as a therapeutic target in the treatment of FS through block or PRF, which has attracted increasing amounts of attention in recent years [11,22].

In addition to metabolic and hormonal changes, the occurrence of primary FS is related to shoulder biomechanical abnormalities and neurological dysfunction. As early as 1959, studies proposed a connection between primary nerve dysfunction and FS, and the authors believed that shoulder nerve stimulation can cause shoulder biomechanical abnormalities and that the two can interact with each other, ultimately resulting in a vicious cycle of pain and shoulder joint dysfunction [32]. This finding also provides evidence that the shoulder nerve can be targeted in the treatment of FS.

However, the nerves innervating the shoulder joint are highly complex and include the SSN, AN, subscapular nerve, lateral thoracic nerve, musculocutaneous nerve, etc., but the largest contributor is the SSN, followed by the AN, and the sensory effects of innervating the glenohumeral joint account for 90% of the innervation [12,13]. The SSN is mainly responsible for the posterior and upper sensations of the shoulder capsule, while the AN is mainly responsible for the anterior and lower sensations of the shoulder capsule [13]. For the above reasons, this study used the SSN and AN as targets for nerve block or PRF treatment in FS patients.

The mechanism of nerve blockage involves interruption of a vicious cycle, which is achieved by injecting corticosteroids and local anesthetics into the periphery of nerves, thereby improving the local blood supply, relaxing muscle spasms, eliminating local inflammation, promoting local tissue metabolism and facilitating joint function recovery. PRF is a high-voltage, low-temperature RF mode formed by discontinuous, pulsed current around nerve tissue. The RF current emitted during each pulse lasts only 20 ms, and there is a 480 ms intermittent period. The heat generated by the RF current near the nerve tissue can fully diffuse during the intermittent period so that the electrode temperature does not exceed 42 °C, thus preventing motor nerve damage [15]. In this study, we chose to use a 10 mm RF trocar at the working end because a longer working end can enable PRF to achieve a greater range of action to achieve better neuromodulation [33]. However, the mechanism of PRF in the treatment of inflammatory pain is still unclear. Hagiwara et al. suggested that PRF may increase the secretion of norepinephrine neurotransmitters and inhibit the 5-hydroxytryptamine pathway, thus alleviating hyperalgesia [34].

In addition, Chen KH [35] reported that the treatment of inflammatory pain by PRF was related to the inhibition of the activation of the c-Jun amino-terminal kinase in the dorsal horn of the spinal cord and the release of excitatory amino acids in the dorsal root ganglia.

This study showed that PRF combined with blocking the SSN or AN in the treatment of FS appears to achieve a better therapeutic effect than nerve block alone, with greater pain relief and better improvement in joint ROM. Most studies [22,36–38] have blocked only the SSN in the treatment of shoulder pain. Alanbay E. et al. [38] randomly assigned patients with hemiplegic shoulder pain caused by stroke to receive PRF or blocking the SSN; 15 patients were included in each group, and the results showed that PRF was superior to nerve block alone for pain relief and ROM improvement at 1 and 3 months after the procedure.

However, there are very few studies on the combination of the SSN and AN for PRF and nerve block treatment. Kim JS et al. [39] reported a case study of a 45-year-old female patient with a 3-year history of calcific tendinitis. She began treatment by receiving two SSN and AN blocks but relapsed 2 weeks later. However, her symptoms were significantly relieved after completing PRF, almost completely disappeared after 3 months, and there was no recurrence after 6 months.

Another study [40] randomized 20 patients with hemiplegic shoulder pain to PRF or nerve block therapy with the SSN and AN at 16 weeks after the procedure. The results showed that pain and joint ROM were significantly improved in both groups compared to before the procedure. The effect of PRF on shoulder pain was similar to that of nerve block, but PRF was superior to nerve block in improving shoulder abduction and external rotation. Regarding technical report, Esparza-Minana JM et al. [17] presented an RF therapy that entailed blocking the SSN and AN, and they suggested that this treatment can block pain conduction in a wider glenohumeral innervation area and achieve a better therapeutic effect.

This study has several limitations. First, this was a single-center prospective cohort study, and it is necessary to conduct a multicenter prospective randomized controlled study to further evaluate the efficacy and safety of PRF and of blocking the SSN and AN. Second, in this study, the SSN and AN were used as targets for PRF and block, while a placebo was not used for evaluation. Finally, compared with patients with primary FS, patients with diabetes, thyroid disease, and hemiplegia after stroke had FS more frequently, more complex pathological characteristics, and more difficult treatment. Therefore, further studies on secondary FS are warranted to evaluate the efficacy of PRF and blocking the SSN and AN.

## Conclusions

PRF or nerve block of both the SSN and AN is effective in patients with primary FS. However, in combination with other treatment options in patients with primary FS, PRF appears to provide better pain relief, more ROM improvement, and better shoulder function recovery than nerve block treatment.

## Supplementary Material

CONSORT_checklist (2).doc

## Data Availability

The data that support the findings of this study are available from the corresponding author, Professor Gang Chen, upon reasonable request.
